# Progress in Surgery

**Published:** 1898-09-17

**Authors:** 


### Progress in Surgery.

### GENERAL SURGERY.

(*Continued from page 412.*)

**Fractures and Dislocations.**—In a paper on the relation of the cause to the immediate and remote results and associated lesions of fractures Dr. R. M. Cunningham[10] remarks that there is usually a combination of the different forms of violence. The most frequent is longitudinal compression and bending, and transverse or oblique compression and torsion. After giving an elaborate classification of the various effects of violence upon the surrounding soft parts, from the periosteum to the skin, he says: "The practical question is, can the limb be saved?" In answering this question, too much stress, he thinks, has been laid upon the visible condition of the wound and the condition of the larger arteries, and too little upon the invisible conditions and the effects of the violence, *per se*. Dr. G. W. Crary[11] presents a clinical report upon the X-rays in fractures. He says that he has been struck with the fact that a well-marked fracture might be present without being shown by the X-rays at all. He brought forward a Röntgen picture from a case of Colles's fracture, in which the fracture could not be discovered even in the negative. The most definite evidence of such fracture had been easily obtained by the surgeons who had examined the case. Where there was no displacement, and the fractured ends were in contact, the picture might not show a fracture at all. This fact, he believed, was of some medico-legal importance. In another case of fractured bones of the forearm, four months after the accident, while there had been distinct and firm bony union, the Röntgen picture had appeared to indicate that union had not taken place. It is well known that these pictures often will not prove that union has occurred unless the callus is old. *Apropos* of a case of fracture of the inferior extremity of the humerus, recently shown by Lucas Championnière[12] at the Academy of Medicine of Paris, in which the cure was perfect and had been effected by massage and mobility from the first; it was said by him that immobility is not an indispensable element, nor even a useful one in the treatment of fractures. A mobilised bone with peripheral massage repairs itself more quickly and more easily than an immobilised one. A great number of fractures of the humerus are amenable to this treatment; all those from the finger to the elbow and those which are above the insertion of the deltoid. Adult and aged subjects are those most benefited by it. Championnière thought that movement never produced pseudarthrosis. Immobility is only useful to avoid deformity. However, in fractures of the humeral or femoral diaphyses, in fractures of the lower end of the tibia, &c., mobilisation cannot be employed. In order to study their results Mr. A. E. Barker[13] has collected 46 cases in which he has operated for fractured bones. They are as follows: Subcutaneous suture of patella for fractures, 19; suture of patella (open method), 10; suture of olecranon for fracture, 6; suture of olecranon for dislocation, 2; suture of bones of forearm (ununited fracture), 2; suture of bones of leg (ununited fracture), 4; suture of clavicle (ununited fracture), 1; suture of patella after division, 2. The results were encouraging, but with care and experience he thought they ought to be better in future, but as far as they went they appeared to justify the belief that operative interference, if done with extreme care, does not involve such risk as to forbid it. To show the importance of retaining and fixing in position all small fragments of bone in fractures, Mr. W. A. Lane[14] describes a simple case of comminuted fracture of the upper end of the radius in a man aged 23. The fragments were secured accurately in position by means of silver wire encircling them and the shaft. Mr. Lane gives skiagraphs of the case before and after the operation. The same surgeon[15] also gives an excellent skiagraph of a case after operation for ununited fracture of the upper end of the tibia. The patient, a man, had broken his leg more than a year before, and had been operated upon previously for the non-union. In this instance Mr. Lane employed three long screws passed in different directions in order to secure perfect apposition and complete immobility of the fragments. The skiagraph was taken about three months after the operation, the result of which was perfect, the patient being able to bear his weight securely on the damaged leg, which he had been unable to do previously.

**Upper Extremity.**—Dr. R. Kennedy[16] describes and illustrates a case of complete atrophy of the deltoid with vicarious restoration of the function. The man, aged 22, sustained a sub-coracoid dislocation of the shoulder joint, which was reduced the same day. The case illustrated the extent to which, on account of the very complete substitution by neighbouring muscles for the function of the atrophied deltoid, recovery took place. This recovery of function may be expected in such a case resulting from injury to the circumflex nerve, without any operative interference. The recovery was so nearly perfect that it was questionable if a better result could have been attained had the nerve been exposed and treated with a successful result. A rare case of fracture of the capitellum of the humerus is recorded by Bastianelli.[17] It occurred in a man aged 35 years, who had fallen down with his arm extended in slight adduction. The patient was treated by complete supra condyloid resection of the humerus, and the fractured articular condyle was found impacted into the anterior part of the inferior humeral epiphysis. After twenty days the constant current was applied with faradic massage, combined with movements of flexion, for two months longer, and after that only occasionally. After this treatment the man was able to work in the fields, and also to shave himself. Only five cases have hitherto been described, one by Hahn and four by Kocher. As regards the signs met with, the existence of exaggerated pronation of the forearm, after an injury to the elbow-joint, with disturbed flexion and extension, may be a symptom of fracture of the capitellum, but only when the epicondyle is felt in the normal position, and a round body having the form and volume of a condyle is found in an abnormal place. J. S. M'Ardle,[18] in a series of cases with skiagraphs, draws attention to the ill-effects of injudicious manipulations of the elbow-joint after displacement backwards of both bones. In the first case, of a woman aged fifty, seven months after dislocation of the elbow and supposed reduction, he cut down on the joint and found the tip of the olecranon in the fossa, and firmly united to it. This fragment being removed by means of a chisel, and the fibroid tissue filling the olecranon fossa and between the fragments by means of a knife, the two freshened ends of the olecranon were fixed together by wire suture. The amount of fibroid tissue filling up the fossa and fixing the bones gave ample evidence of the rigorous procedures adopted to restore the bones to place, even after the olecranon had given way. The result was everything that could be desired, motion being unrestricted by the eighth week. In a second case of a boy aged 13 years, with an unreduced backward dislocation of the elbow (five months after the injury), M'Ardle divided the olecranon through its base, dissected out the fibroid material, fixing the bones in the abnormal position, restored the bones to their normal position (after section of the triceps tendon), and wired as in the previous case. Exactly two months after the operation the bones were in perfect position and outline, and union by bone complete. He thought a study of X-ray pictures would prove of great value in judging of the proper time for allowing movement after operations on bone, for by the photographs shown it was seen how different the course of repair is in the child and in the adult—only a faint line of bony union appears in the adult bone after six weeks, while in the child union is quite complete in the eighth week. In the treatment of Colles's fracture of the radius, E. A. Tracy,[19] after reduction, moulds a piece of wood fibre splint material over the dorsum of the forearm and hand in a semi-prone position, the hand in line with the forearm. A bandage is then applied over the splint. This does not interfere with passive motion of the fingers, which is commenced on the first day, and is readily removed and reapplied to permit of passive motion of the wrist-joint, which is commenced on the fifth day. The splint is moulded directly over the skin, and requires no padding or compress. It is discarded on an average of about 21 days. By means of the X rays Dr. H. M. Silver[20] has seen in a lad of 16 an epiphysial separation of the lower end of the radius, combined with a fracture of the base of its styloid process. There was also a fracture of the styloid process of the ulna, which was carried downwards towards the pisiform bone. Under an anæsthetic the hand was carried forcibly inwards to overcome the slight outward displacement. Another skiagraph, taken eight and a half months later, showed the radial epiphysis in its new position, some slight thickening of its lower extremity, and the styloid process of ulna united. Dr. R. H. Shaw[21] records a case of compound dislocation of the ungual phalanx of the thumb in a boy aged 11 years. The proximal end protruded through an opening about an inch in length in the skin on the palmar aspect. The dislocation was reduced, and the wound, which was dressed antiseptically, healed slowly with impaired movement in the joint.

[10] Charlotte Med. Jour., Dec., 1897, p. 661. [11] New York Med. Jour., May 7, 1898, p. 654. [12] New York Med. Rec., May 7, 1898, p. 658. [13] Lancet, Aug. 20, 1898, p. 467. [14] Clinical Jour., April 20, 1898, p. 471. [15] Clinical Jour., June 13, 1898, p. 147. [16] Brit. Med. Jour., June 11, 1898, p. 1,513. [17] Amer. Medico-Surgical Bulletin, Mar. 10, 1898, and La Sett. Medica, No. 11, 1897. [18] Dublin Jour. Med. Sci., April 1, 1898, p. 284. [19] Jour. Amer. Med. Assoc., Nov. 20, 1897, and Therapeutic Gaz., Feb., 1898, p. 131. [20] New York Med. Jour., May 7, 1898, p. 654. [21] Lancet, May 14, 1898, p. 1,259.

